# pH-Responsive Hydrogels: Recent Advances in Pharmaceutical Applications

**DOI:** 10.3390/polym17111451

**Published:** 2025-05-23

**Authors:** Georgia Patroklou, Efstathia Triantafyllopoulou, Paraskevi-Evelina Goula, Vasiliki Karali, Maria Chountoulesi, Georgia Valsami, Stergios Pispas, Natassa Pippa

**Affiliations:** 1 Section of Pharmaceutical Technology, Department of Pharmacy, School of Health Sciences, National and Kapodistrian University of Athens, Panepistimioupolis Zografou, 15771 Athens, Greece; georgia98pat@gmail.com (G.P.); efstrian@pharm.uoa.gr (E.T.); goulaevelina@gmail.com (P.-E.G.); basilikekarale@gmail.com (V.K.); mchountoules@pharm.uoa.gr (M.C.); valsami@pharm.uoa.gr (G.V.); 2 Theoretical and Physical Chemistry Institute, National Hellenic Research Foundation, 48 Vassileos Constantinou Avenue, 11635 Athens, Greece; pispas@eie.gr

**Keywords:** pH-responsive, hydrogels, drug delivery, polymers, wound healing, chitosan, poly(acrylic acid), controlled release, anticancer therapy

## Abstract

Hydrogels are three-dimensional polymeric systems, being able to accommodate different categories of bioactive agents and act as promising drug delivery systems in many different biomedical applications. Due to their extended 3D network, hydrogels exhibit many advantages, such as extensive loading capacity and controlled drug release profiles, combined with characteristics such as biocompatibility and biodegradability, due to their constructive polymeric biomaterials. Moreover, hydrogels are capable of being administered via different routes of administration, including systemic and topical ones, due to their tunable characteristics. Stimuli-responsive hydrogels are characterized as smart biomaterials, while environmental stimuli, such as pH, can be employed to trigger on-demand drug release from the hydrogels via the provocation of conformational changes. In the present study, an emphasis on the pH-responsive hydrogels is taking place through various literature cases in drug delivery, wound healing, and some alternative applications, including implantation, oral administration, etc., wherein many different polymeric derivatives have been utilized. Moreover, the role of each used polymer or polymeric combination with other functional biomaterials, their mode of structure formation (for example, crosslinking), and their content release mechanism are highlighted, as well as the therapeutic effect of the hydrogels on different pathological conditions, as promising candidates for pharmaceutical applications.

## 1. Introduction

**Scheme 1:**
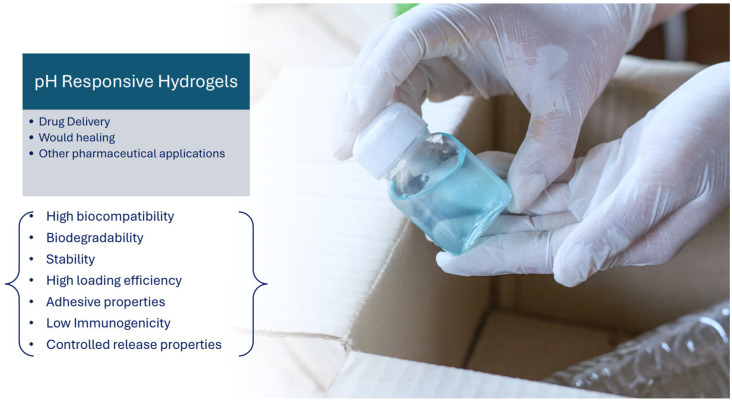
The pH-responsive hydrogels.

Hydrogels are three-dimensional polymeric systems formed through chemical bonds (covalent bonds) and physical interactions (e.g., hydrogen bonds, Coulombic forces, Van der Waals, coordination bonds, etc.) [1,2,3,4]. Drug molecules, bioactive chemicals, natural goods, nutritional additives, and biomolecules are just a few of the many content categories that can be loaded into hydrogels. In addition to their tunable solubility, high water absorption capacity, and moisture retention [3], hydrogels’ extended 3D interior surface gives them a high loading/encapsulation efficiency, making them perfect for drug delivery applications. They also have the potential to hold large amounts of water molecules through capillary action and osmotic pressure, making them resemble the extracellular matrices of living tissues [4]. Their main advantages include a profile of high safety with low immunogenicity, high biocompatibility, biodegradability, and cytocompatibility due to the sustainability of their used constructive polymeric biomaterials, along with some structural characteristics such as mechanical stability, high loading capacity, controlled release ability, adhesive properties, and tunable chemical structure that can be modified towards enhancing targeting ability and triggered content release (Scheme 1) [5]. However, some of their drawbacks include some scale-up limitations, such as batch-to-batch variability and fragile mechanical strengths, along with some biodegradation phenomena that should be under continuous control, such as catabolization, gelation, and microbial instability [6,7].

Stimuli-responsive hydrogels can be considered as smart biomaterials, while the external triggers and environmental variations, such as pH, temperature, electrical and magnetic fields, light, and biomolecule concentration such as elevated ROS levels, high glucose concentrations, and overexpressed enzymes, can be employed to trigger on-demand drug release from the hydrogels.

From a biological point of view, variations in pH are known to take place at several body sites, such as the gastrointestinal tract, vagina, and blood vessels, which can provide a suitable condition for pH-responsive drug release, as well as within intracellular compartments, such as endosomal/lysosomal, and also in cases of pathological conditions, such as oxidative stress and tumor microenvironment. pH-responsive hydrogels are capable of being designed and customized to exhibit a site-specific response to particular pH ranges, enabling controlled drug release in these specific pH environments via pH-dependent swelling or collapse behavior [7]. pH-responsive hydrogels are composed of polymeric backbones with ionizable functional groups [8,9]. These ionizable groups can achieve a targeted and regulated drug release in response to local pH alterations by exhibiting conformational or solubility changes or hydrogel transitioning between swelling and deswelling states. Some applications of the pH-responsive hydrogels already mentioned are gastroretentive drug delivery, intestine and colon targeting drug delivery, their utilization in protein or peptide delivery, in transdermal patches, and for cancer targeting, with the aim of enhancing treatment effectiveness and preventing side effects [8,9].

**Figure 1:**
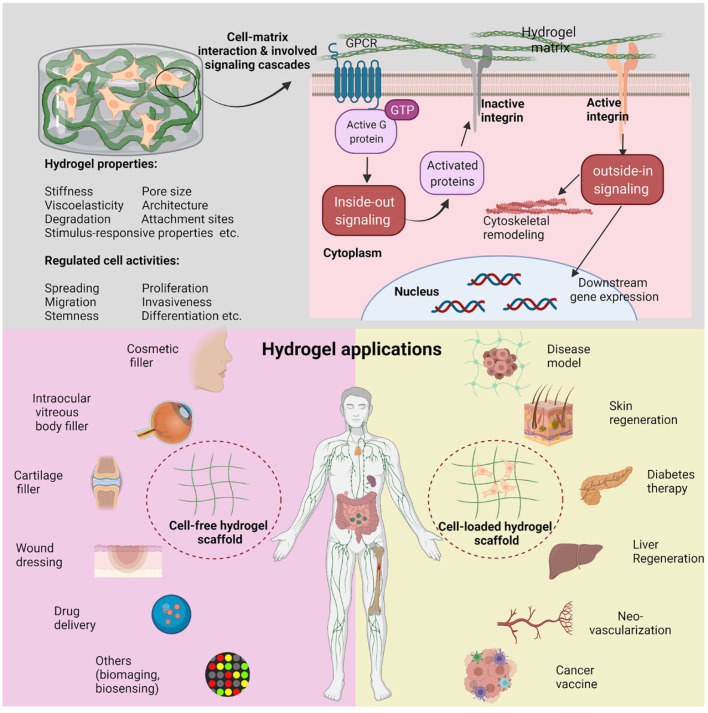
Schematic images for indicating the interactions between cell and hydrogel matrix, uncovering the influences of hydrogel physicochemical properties on cell biology via correspondingly triggering signaling cascades (e.g., inside-out and outside-in signaling), and illustrating various hydrogel biomedical applications of cell-free and cell-loaded hydrogels. (Adapted from [10]).

In the present study, emphasis is given to the application of the pH-responsive hydrogels, providing a variety of literature studies in drug delivery, wound healing, and some alternative applications, including implantation, oral administration, etc., by examining cases comprising many different polymeric candidates [7]. Namely, a detailed description of different pH-responsive hydrogels is presented, highlighting the role of each polymer utilized or of the combination of different polymers or the combination with other functional biomaterials, their mode of structure formation (for example, crosslinking), their content release mechanism, as well as their therapeutic effect on different pathological conditions, as promising candidates for biomedical applications (Figure 1).

## 2. Methodology

The literature search and review were conducted via the electronic databases PubMed, Google Scholar, and Scopus. The publications from the past six years (2019–2025) were used for the systemic investigation of hydrogels’ applications. The literature search was performed, excluding the studies with publication language other than English, conference abstracts, or no full text availability.

## 3. Recent Advances in pH-Responsive Hydrogels

### 3.1. Drug Delivery

A dual pH- and thermo-responsive hydrogel based on poly (acrylic acid) and a functionalized chitosan is fabricated for controlled drug delivery to the colon. Chitosan is easily degraded only by enzymes and bacteria in the colon but can also undesirably swell in the stomach and release the Active Pharmaceutical Ingredient (API). Poly(acrylic acid) is used because of its ability to suppress the swelling of chitosan in the stomach and promote targeted drug release. The hydrogel is pH dependent due to the Schiff-base reaction formed between the amino groups and the aldehyde groups of the polymers. The highest drug release occurs at pH 7.4 due to the breaking of these bonds. Biomaterials are encapsulated intact due to the usage of the Diel-Alder click reaction and the Tz-click reaction, while the nitrogen—which is released from this reaction increases the porosity of the hydrogels, the drug loading contents and the release [11].

**Figure 2:**
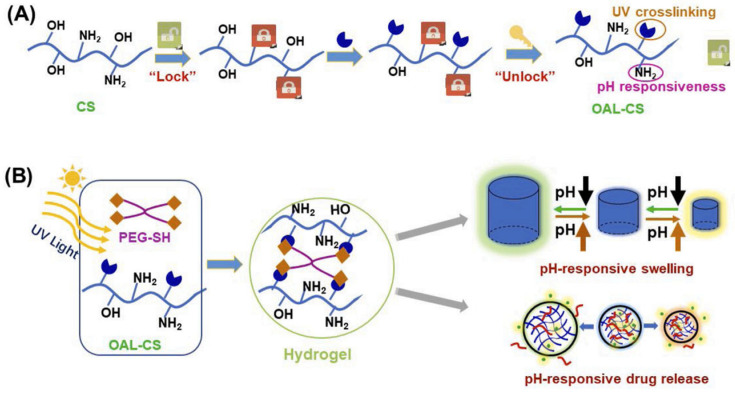
Schematic synthesis of pH-responsive UV-crosslinkable C6 O-allyl chitosan (OAL-CS) and multi-functional properties of OAL-CS hydrogel. (**A**) Synthesis procedure of OAL-CS via protection/deprotection of amino groups. The amine groups (–NH2) were “Lock” to prevent them from participating in the next reaction. After UV-crosslinkable groups were attached to CS, amine groups (–NH2) were “Unlock” to recover CS’s pH responsiveness; (**B**) the OAL-CS hydrogel was formed via UV-triggered “thiol-ene” click chemistry in which 4-arm PEG-SH served as a crosslinker. The hydrogel had reversible pH responsiveness and pH-triggered drug control release. (Adapted from [12] with permission).

A doxorubicin-loaded hydrogel comprised of a chitosan derivative, namely O-allyl chitosan (OAL), was formulated so that the low water solubility of chitosan would be increased over a wide pH range without reducing the molecule’s amino groups (Figure 2). This ensures that the hydrogel retains its pH-responsive capability. Four-arm polyethylene glycol (PEG-SH) is used as a crosslinking agent to enhance the mechanical strength of chitosan and enable rapid gel formation. The hydrogel is synthesized through a UV-triggered “thiol-ene” click chemical reaction between the thiol groups of polyethylene glycol and the allyl groups of the modified chitosan. At pH 6.8, the release of doxorubicin is higher, while in an acidic environment, the amino groups of the hydrogel interact with the deprotonated carboxyl groups of the API, resulting in minimal drug release. Meanwhile, the amino groups of BSA become protonated, leading to the formation of electrostatic repulsive forces that cause hydrogel swelling and subsequent drug release. In conclusion, this hydrogel can serve as a targeted drug delivery carrier, enabling controlled drug release based on the pH of the target tissue [12].

Prunus armeniaca gum (PAG) is combined with acrylic acid (AA) to synthesize a pH-responsive hydrogel, which can be used as a controlled drug delivery system. AA possesses remarkable water retention capability and can swell at pH 7.4 due to the deprotonation of its carboxylic acid groups, leading to the dissociation of the hydrogel structure and thus the drug release. PAG has emulsifying, stabilizing, binding, and antioxidant properties. The hydrogel is synthesized through free radical polymerization using N,N-methylene bisacrylamide as a crosslinking agent and potassium persulfate as an initiator. Tramadol hydrochloride is used as a model drug to demonstrate the hydrogel’s capability for drug release in specific sites [13].

Qian et al. (2019) [14] formed an injectable, self-healing, and pH-responsive hydrogel. The hydrogel is fabricated using N-carboxyethyl chitosan (CEC), a water-soluble derivative of chitosan, and aldehyde hyaluronic acid (A-HA), which imparts pH-responsive properties due to the aldehyde groups that are able to form Schiff bases with other reactive groups of polymers. Hyaluronic acid is also known for its excellent biocompatibility and biodegradability. The CEC/A-HA hydrogel is used for the release of doxorubicin, which shows a cytotoxic effect on HeLa cells. The drug release is higher at pH 7.4 due to the deprotonation of carboxyl groups, leading to the formation of electrostatic repulsive forces and increased hydrogel swelling. At pH 5.8, amine groups are also deprotonated, but swelling remains limited due to the hydrogen bonds formed between the modified polymers, which restrict hydrogel expansion and degradation. The biocompatible and pH-responsive CEC/A-HA hydrogel can be a promising candidate for sustained drug release in tumor therapy [14].

Another pH-sensitive hydrogel is composed with the aim of controlled and targeted release. For this purpose, chitosan is used, and it is crosslinked with 8-aldehyde-7-hydroxy-4-methyl coumarin, aiming to minimize toxicity and maximize efficacy. Coumarin also possesses antioxidant, antibacterial, and anticancer properties. The injectable hydrogels are prepared via the formation of Schiff base bonds and hydrogen bonds between the polymers responsible for pH responsiveness and self-healing ability, while taxifolin is incorporated into the hydrogel matrix. Even though the drug release is suppressed in an alkaline environment, in an acidic environment Schiff base bonds between amine and aldehyde groups become unstable due to the electrostatic repulsive interactions between the protonated amine groups of chitosan, leading to the swelling of the hydrogel and the release of the API [15].

Bacterial cellulose (BC) and chitosan (CS) are used as the components of a pH-responsive hydrogel through the “one-pot synthesis method”. BC exhibits excellent mechanical properties, water retention capacity, and high biocompatibility. Chitosan, as it is already known, has good biocompatibility and antibacterial activity, but it has challenging usage properties as well, such as poor mechanical properties and low water solubility. BC and CS are linked via hydrogen bonds to form a dense microstructure that enhances the hydrogel’s mechanical properties. The mechanical characteristics can be enhanced by modulating the BC content. The hydrogel is successfully used as a drug delivery carrier, loaded with naproxen, while the drug release is higher in alkaline conditions [16].

The dual pH/glutathione (pH/GSH)-responsive hydrogel TCS/PUE is a combination of thiolated chitosan (TCS) hybridized with puerarin (PUE). TCS is a water-soluble chitosan due to the hydrophilic thiol groups in its structure. These groups also provide strong adhesion and penetration abilities through the formation of intra- or inter-disulfide bonds, as well as good gelling properties and contractility in physiological pH environments. Moreover, puerarin can self-assemble into hydrogels without further chemical modification. It has anti-inflammatory properties, can prevent vascular relaxation, and improves cardiac function. Berberine is selected as a model API, and the protonation of TCS under acidic conditions (pH 5.8) leads to the hydrogel’s degradation and the API’s release. This confirms that the TCS/PUE hydrogel is a promising drug delivery carrier having the capability for controlled release in a pH-dependent manner [17].

CS-NSA/A-HA is a chitosan-based hydrogel (CS) modified with nitrosalicylaldehyde (NSA) and aldehyde-functionalized hyaluronic acid (A-HA) through Schiff base crosslinking. CS contains numerous amino and hydroxyl groups, enabling it to crosslink with other polymers and enhancing drug penetration via the epithelial cells. NSA has anticancer properties due to the presence of -NO_2_ groups and can form Schiff base bonds with the reactive -NH_2_ groups of the polysaccharides. A-HA can develop chemical crosslinks with amino groups via Schiff base formation, improving the hydrogel’s stability and drug-loading efficiency.

This hydrogel is used for the targeted co-delivery of cisplatin and doxorubicin. The authors demonstrated enhanced inhibition of A549 lung cancer cells compared to the individual drug carriers. Both APIs exhibit the highest release at pH 5.5—that corresponds to the tumor microenvironment, because under acidic conditions the hydrogel’s electrostatic and covalent bonds break down, leading to the degradation of its network and controlled drug release [18].

Poly(vinyl alcohol) (PVA)-based hydrogels are widely used as drug delivery carriers. However, they are impaired by water instability and lack of pH sensitivity due to the absence of ionizable functional groups. To address these limitations, PVA is crosslinked through esterification reactions with citric acid, which introduces carboxyl groups to the structure. These functional groups enhance biocompatibility, can regulate polymer hydrophilicity, and transfuse pH-responsive and antibacterial properties, while they can increase the number of available sites for bioconjugation. The swelling behavior of the hydrogel is influenced by pH and the citric acid’s concentration. Namely, maximum swelling and, consequently, maximum drug release occur at pH 7.4 due to the deprotonation of carboxyl groups of the crosslinking agent, whereas higher swelling is observed at lower citric acid concentrations due to an increase in the hydrogel’s porosity. The release of the incorporated API, ciprofloxacin, is prolonged by embedding silver nanoparticles into the hydrogel. The supramolecular structure slows down hydrogel swelling and drug diffusion, transforming the system into a sustained drug delivery platform [19].

Arjama and colleagues (2022) combined hyaluronic acid (HA), dopamine (DOP), chitosan (CS), and polyethylene glycol (PEG) to form cubic particles of the hydrogel (HA-DOP-CS-PEG)5 using the layer-by-layer technique. Doxorubicin is incorporated into the hydrogel through ionic interactions with the HA-DO polymer. Moreover, the multiple-layer hydrogel’s network allowed increased drug loading. In an acidic environment with varying concentrations of glutathione, there is a steady increase in drug release, showing the highest release at pH 5.0. This development exhibited controlled drug release and improved therapeutic effectiveness, avoiding the systemic toxicity observed with other administration methods. Additionally, it can be utilized for chemotherapy/phototherapy of breast cancer due to its effectiveness in vitro [20].

Citric acid (CA) is used as a cross-linking agent between quince and the polyanionic pectin for the formation of hydrogel sponges. The use of the green chemical CA enhances mechanical stability and ensures the prolonged release of the drug. β-Cyclodextrin is an amphiphilic oligosaccharide, which can improve hydrophobic APIs properties by incorporating them into its hydrophobic core. As a result, it can be used in the preparation of copolymerized drug delivery systems. The selected API is domperidone, a dopamine D2 receptor antagonist with antiemetic, galactagogue, and gastroprokinetic properties. The carboxylic acid groups within the matrix are protonated at high pH, generating anions that increase the repulsion of polymer chains, leading to network swelling. This hydrogel is used as a controlled drug delivery system in a basic environment, where swelling is at its maximum. Thus, it contributes not only to minimizing gastrointestinal side effects but also to improving patient compliance and therapeutic efficacy [21].

A hydrogel network was formed by a traditional Chinese herb with high therapeutic value and a modified cellulose (MSCCMC). The carboxyl groups of MSCCMC deprotonate by pH increase, generating electrostatic repulsive forces that expand the hydrogel matrix. The pH-responsive MSCC-MSCCMC hydrogel is successfully used for the incorporation and release of Lactobacillus probiotics in the gastrointestinal tract, protecting them from degradation in the acidic environment or by digestive enzymes and bile salts. These bacteria are used for the treatment of hypercholesterolemia due to their bacteriostatic properties and their ability to lower blood cholesterol levels [22].

Quercetin has a significant anticancer effect but poor physicochemical properties for oral administration. For this purpose, a quercetin-loaded hydrogel is developed through the formation of ionic bonds between the carboxyl groups of the anionic polysaccharide gellan gum and the calcium ions of the CaCl₂ solution to enable the effective delivery of quercetin in the gastrointestinal tract. The hydrogel is formulated using polysaccharides, which are chosen for their low toxicity, high swelling capacity, and the presence of multiple functional groups that can interact via hydrogen bonding with mucin, enhancing the formulation’s retention time in the gastrointestinal tract. These carboxyl groups provide pH-responsive properties as well. At basic pH, water absorption, hydrogel swelling, and ultimately, quercetin release are promoted due to negative electrostatic interactions in the hydrogel. The release is also improved by degradation of the hydrogel network by colonic enzymes [23].

Due to the several assets of chitosan, it is the polysaccharide of choice for a lot of innovative developments. In this case, a hydrogel loaded with fluorouracil is fabricated for targeted and controlled release into the colon. To prepare the hydrogel, chitosan is crosslinked with 2-acrylamido-2-methylpropane-sulfonic acid (AMPS) and acrylic acid (AAc). The swelling of the hydrogel is minimal in an acidic environment due to the formation of intramolecular hydrogen bonds (SO_3_^−^–NH_3_^+^) between the deprotonated sulfonic groups of AMPS and the chitosan’s protonated amine groups. Conversely, swelling reaches its maximum at pH 4–7 due to intramolecular repulsive forces caused by the deprotonation of chitosan’s amine and hydroxyl groups, as well as the deprotonation of AAc carboxyl groups [24].

Recently, a self-healing, injectable, and pH-responsive drug delivery system was designed for the localized treatment of breast cancer by Li et al. (2024) [25]. The hydrogel is based on a derivative of chitosan (CMCS), which offers improved water solubility, biodegradability, stability, biocompatibility, and functionality. Polyaldehyde gum arabic (OGA), the oxidized form of the natural polysaccharide, is characterized by multiple aldehyde groups enabling cross-linking and providing pH-responsiveness as well. The hydrogel’s swelling capacity is inversely proportional to the concentration of the OGA. Within this network, graphene oxide (GO) and doxorubicin are incorporated. GO enhances the hydrogel’s mechanical strength and effectiveness, while it offers good colloidal stability, surface modification ability, and biocompatibility. The drug release is significantly higher in the tumor microenvironment compared to physiological pH, raising the hydrogel to an ideal carrier for the controlled and targeted release of anticancer APIs [25].

The CS-DA/OP hydrogel is a mucoadhesive, pH-responsive hydrogel designed for the localized drug delivery for the treatment of colorectal cancer and/or bacterial infections. Namely, chitosan (CS) is conjugated with dihydrocaffeic acid (DA), and the molecule was further crosslinked with oxidized pullulan (OP) to form the CS-DA/OP hydrogel. The aforementioned mucoadhesive properties are owed to DA, which is binding to both organic and inorganic surfaces. This feature minimizes unintended drug diffusion, thereby reducing potential toxicity during drug administration. Afterwards, the hydrophobic anticancer drug doxorubicin and the hydrophilic antibacterial API amoxicillin were incorporated into the hydrogel. When exposed to an acidic environment, such as that of a tumor or an infected wound, the hydrogel exhibits a high swelling ratio and increased pore size, leading to an enhanced release of the embedded APIs [26].

Fathi and colleagues (2019) formulated a PNIPAAm-co-IA hydrogel for the localized delivery of chemotherapeutic agents such as doxorubicin (DOX). The hydrogel is comprised of chitosan and glycerophosphate (CS/GP) as well. The CS/GP matrix acts as a thermosensitive agent that prolongs drug release and serves as a local drug delivery system. PNIPAAm is incorporated into this network to ensure a sharp phase transition above the lower critical solution temperature (LCST) at body temperature, enabling drug release in response to pH changes. The addition of small amounts of diprotic acids, such as itaconic acid (IA), imparts pH-responsive properties to the drug delivery system. Indeed, at an acidic pH, the protonation of CS amino groups leads to increased water diffusion into the hydrogel, resulting in greater swelling and subsequent selective release of the anticancer API [27].

Succinoglycan dialdehyde (SGDA) and hydrazine-functionalized alginate (HZ-Alg) were amalgamated to form a pH-sensitive hydrogel used for controlled drug delivery and tissue engineering applications. SGDA acts as a cross-linking agent that enhances the durability and thermal stability of gelatin-based hydrogels. Within the hydrogel network, a reversible hydrazone bond is formed, endowing the hydrogel with pH-responsive properties and self-assembly capability. The hydrogel successfully enables the targeted release of the anticancer API, 5-fluorouracil, in acidic environments, such as the tumor microenvironment [28].

Succinoglycan dialdehyde/aminoethylcarbamoyl-β-cyclodextrin (SGDA/ACD) is a pH-dependent hydrogel due to the properties of SGDA. SGDA also provides thermal and mechanical stability. Baicalein is utilized due to its antioxidant, antibacterial, anti-inflammatory, and anti-HIV activity. It is a promising drug for Alzheimer’s and Parkinson’s disease with low bioavailability due to low water solubility. ACD managed to increase the solubility of baicalein and improve the pH-responsiveness of the hydrogel. The drug release is higher at pH 7.4 because of the deprotonation of the carboxyl groups of SGDA and the electrostatic repulsive forces that are created, as well as the disintegration of the hydrogel due to the hydrogen bonds’ destruction. This hydrogel is used for the controlled delivery of the hydrophobic API directly to the small intestine, increasing its pharmacokinetics [29].

### 3.2. Wound Healing

In this section, the pH-responsive hydrogels that are used for wound healing will be presented. The main class of polymers that are used for the design and development of pH-responsive hydrogels for wound healing are the natural biopolymers such as chitosan and gelatin (Table 1). Hydrogels prepared based on chitosan (CS) have excellent application in wound healing due to their antibacterial properties. However, the use of CS is limited because of its low water solubility; therefore, its hydrophilic derivatives are used. Such an example is the carboxyethyl chitosan, which is combined with polyethyleneimine (PEI) via the crosslinking agent oxidized pectin (OP) to create a hydrogel of improved mechanical properties and enhanced antibacterial capability. The pH sensitivity of the hydrogel is the result of Schiff base bonds formed between its polymers. These bonds also provide self-healing ability. The hydrogel degrades rapidly in an acidic environment due to the breakdown of Schiff base bonds, and the antimicrobial properties of CS and PEI can be utilized for the healing of skin wounds. Studies are also being conducted on its use in artificial skin and wearable devices [30].

Another interesting derivative is quaternized chitosan, which is used because of its satisfactory water solubility across a broad pH range and the protonated amine groups in its structure, which increase the positive charge of the hydrogel and facilitate the destruction of negatively charged bacterial cell membranes. In this case, a photosensitizer is embedded in the hydrogel, which can induce permanent bacterial microorganism destruction through Near-Infrared (NIR) laser-induced photothermal therapy. Additionally, a crosslinking agent is used: oxidized hyaluronic acid, which strengthens the hydrogel network and provides self-healing ability through its aldehyde groups. The reversible chemical bonds—such as Schiff base bonds—formed enrich the hydrogel with pH-responsive capability as well. The hydrogel exhibits extended release of berberine in a slightly acidic environment due to the protonation of the carboxyl groups of oxidized hyaluronic acid (OHA), reducing the electrostatic interactions between the structural polymers and the positively charged API. In this manner, this hydrogel can be used as a wound dressing to combat bacterial infection without the need for antibiotics [31].

A double-grafted chitosan-based hydrogel is developed using dual dynamic Schiff base and phenylboronic ester bonds. Its primary goal is to promote the healing of sports-related diabetic foot wounds by reducing inflammation and enhancing angiogenesis. Dihydrocaffeic acid (DA) possesses strong antioxidant properties and forms Schiff base bonds with the amino groups of chitosan (CS), producing the CS-DA polymer. Due to its catechol groups, DA imparts adhesion capabilities to the hydrogel, further reinforced by incorporating the cationic amino acid L-arginine. This system ensures strong wound adhesion. Benzaldehyde-functionalized polyethylene glycol-co-poly(glycerol sebacic acid) (PEGS-BA) forms Schiff base bonds via its aldehyde groups with CS, while phenylboronic acid (PBA) establishes phenylboronic bonds with the catechol groups of DA. These interactions confer pH responsiveness to the hydrogel, making it multifunctional. Additionally, the hydrogel incorporates metformin, and it is coated with polydopamine-coated reduced graphene oxide (rGO@PDA) to improve its mechanical strength. Drug release is higher in acidic environments, such as inflamed wounds, due to the breakdown of the bonds that maintain the hydrogel network [32].

A catechol-grafted chitosan is selected for the formation of a hydrogel loaded with curcumin to be used as a preventive mechanism for bacterial infections via the reduction of inflammation and the promotion of infected wounds’ recovery caused by *S. aureus*. Namely, 3,4-dihydrocinnamic acid is linked to chitosan so as to increase the mechanical stability of the structure. Due to its hydrophobicity, curcumin is encapsulated into inorganic nanospheres through the formation of a coordination bond between the carbonyl group of curcumin and the Fe^3+^ ions, which endows the hydrogel with pH-responsive properties. The strong and reversible bonds between the catechol groups of the polymer and the metal ions Fe^3+^ in the hydrogel structure allow for the controlled and pH-dependent release of the bioactive compound, enabling wound healing [33].

Another chitosan-based hydrogel is selected as a wound dressing for the release of APIs in an alkaline environment. The cationic polymer is combined with the anionic polymer γ-glutamic acid via electrostatic interactions, which are influenced by the pH of the environment, to form a polyelectrolyte complex aimed at delivering the API for wound healing. An amino acid, β-Ala, is also incorporated into the hydrogel, which acts as a crosslinking agent between the polymers, aiming to stabilize the network. β-Ala also contributes to the incorporation of positively charged APIs, forming neutral complexes under alkaline conditions. The API used is benzalkonium chloride with antimicrobial properties. At an acidic pH of 4.09–6.50, the electrostatic interaction between the polymers is very strong, so that swelling is minimal. As the pH increases, the swelling capacity increases accordingly because the electrostatic interactions become weaker. The authors declared the capability of this hydrogel as a dressing for the healing of chronic wounds [34].

A pH/ROS dual-responsive hydrogel is a promising wound dressing for the healing of chronic diabetic wounds and possesses antibacterial properties. Namely, ε-polylysine (EPL), which is a natural antimicrobial polypeptide with excellent biocompatibility and antibacterial action, is conjugated with caffeic acid via crosslinking, leading to the creation of polymer CA-EPL (CE). The catechol groups of this polymer enable the creation of reversible covalent ester boronic bonds, making it a suitable substrate for the preparation of hydrogels in the form of dressings. Phenylboronic acid (PBAs) is also used for its ability to form covalent ester boronic bonds with diols and its sensitivity to ROS. PBA is conjugated with oxidized dextran (OD) to form the polymer POD. The two polymers, CE and POD, are crosslinked between the amino groups of CE and the aldehyde groups of POD. The hydrogel incorporates diclofenac sodium and mangiferin (MF), which is initially encapsulated in micelles (MIC). In the infected diabetic wound, an acidic environment prevails, which breaks the Schiff base bonds holding the hydrogel’s structural entities, leading to the destruction of the network and the initial release of the API. Then, due to the highly acidic environment during the healing process, the micelles are broken, releasing their cargo as well [35].

Gelatin (Gel), a highly biocompatible macromolecule that is non-immunogenic, inexpensive, and with controlled biodegradability, is used as a base for the development of a wound healing system in the form of a hydrogel. For the preparation of the pH-responsive hydrogel, ethylenediamine-modified gelatin is mixed with oxidized dextran. Zinc oxide nanoparticles are incorporated into the hydrogel. Furthermore, paeoniflorin is loaded in micelles, and afterwards the micelles were integrated into the hydrogel. This system combines hemostatic, antibacterial, adhesive, and angiogenic properties. In general, an infection leads to a decrease in pH and an increase in ROS concentration in the environment, which favors the release of ZnO nanoparticles by the breakdown of Schiff base bonds and the disintegration of micelles, leading to the API’s release. This hydrogel is deemed an excellent choice for local drug delivery, particularly for treating chronic diabetic bacterial wounds [36].

A hydrogel used as a wound dressing for bacterial infections without antibiotic resistance is formed by Du et al. (2023) [37] with the aim of API controlled release. The hydrogel, composed of gelatin methacryloyl (GelMA) and hyaluronic acid-aldehyde (HA-CHO), is prepared through photo-crosslinking, showing self-healing capability. The swelling ability and porosity of the hydrogel are a function of the HA-CHO polymer’s concentration. A high concentration of HA-CHO in the structure leads to an increase in hydrophilic groups and water diffusion into the hydrogel, resulting in maximum swelling. The drug delivery system incorporates gentamicin and lysozyme. Due to the disrupted Schiff base bonds and electrostatic interactions created in an acidic environment, the release of the APIs is maximized at pH 5.0 [37].

**Table 1:** pH-responsive systems for wound healing.

Reference	Hydrogel	API	Added Value
[32]	PEGS-PBA-BA/CS-DA-LAG (PC) dihydrocaffeic acid and L-arginine cografted chitosan (CS-DA-LAG) phenylboronic acid and benzaldehyde bifunctional polyethylene glycol-co-poly(glycerol sebacic acid) (PEGS-PBA-BA)	Metformin (Increase of sensitivity of peripheral local cells to insulin).	Healing of athletic diabetic foot wounds and provision of a local-specific drug dual-response release strategy for the treatment of type II diabetic feet.
[35]	DS&MIC@MF Diclofenac sodium (DS) Encapsulated Magniferin (MF) in hydrophobic core of micelles (MIC)	Diclofenac sodium (DS) Mangiferin (MF)	Wound dressing for promoting the repairing of chronic diabetic wounds.
[37]	GelMA/HA-CHO/GS/LZM gelatin methacryloyl (GelMA) hyaluronic acid-aldehyde (HA-CHO) Gentamicin Sulfate (GS) Lysozyme (LZM) poly(styrene-b-(ethylene-co-butylene)-b- styrene) (SEBS)	Gentamicin Sulfate (GS) Lysozyme (LZM)	Wound dressing for bacterial infections without bacterial resistance and controlled drug release.
[38]	GCM Curcumin (CUR) Hydrogen (H_2_) Benzaldehyde-modified Pluronic F127 polymer (FCHO) Gelatin (Gel) Magnesium-based micromotors (Mg-micromotors) Gel solution + CUR-FCHO + Mg micromotors = GCM hydrogel	Curcumin (CUR) Antibacterial and Antioxidative Activities. Hydrogen (H_2_) Antioxidant activity Anti-Inflammatory activity	Promotion of healing of diabetic wounds by reducing the Inflammation of the wound and scavenging the ROS.
[30]	CEC/OP/PEI Carboxyethyl Chitosan (CEC) Oxidized Pectin (OP) Polyethyleneimine (PEI)	Chitosan (CS) Antimicrobial properties and antioxidant activity. Polyethyleneimine (PEI) Antimicrobial properties	Wound dressings for the healing of skin defects in clinical applications. In artificial skin and wearable devices.
[39]	Peptide-PNIPAm@ORO Amphiphilic pentapeptides (APPs) Thermosensitive polymer (PBIPAm) Origanum Oil (ORO)	Amphiphilic pentapeptides (APPs) Antibacterial Activity Origanum oil (ORO) Antibacterial essential oil.	Wound angiogenesis and cell proliferation accelerate the formation of wound granulation tissue, re-epithelialization, and collagen remodelling thereby speeding up the healing process of infected wounds. Eradication of biofilm and promotion of the healing of infected wounds provides a promising strategy for the healing of clinical chronic wounds.
[7,40]	GelMA/OSSA/PMB Gelatin methacryloyl (GelMA) Polymyxin B (PMB) Oxidized sulfated sodium alginate (OSSA)	Polymyxin B (PMB) Against MDR Gram-Negative Organisms Oxidized sulfated sodium alginate (OSSA) Ameliorates oxidative stress and reduces inflammation after eradicating MDR gram-negative infection.	Wound healing by reducing inflammation and promoting vascularization and re-epithelialization.
[31]	QCS/OHA-PEDOT-BBH-EGF Quaternized chitosan (QCS) Oxidized hyaluronic acid (OHA) Pol (3, 4-ethylenedioxythiophene):poly(styrene-sulfonate) (PEDOT:PSS) Berberine (BBH) Epidermal growth factor (EGF)	Chitosan (CS) Inherent antibacterial ability due to their hydrophilic polycationic structure. Quaternized chitosan (QCS) Antibacterial/hemostatic properties due to the protonated amine group. Adsorb negatively charged neuraminic acid on the surface of blood cells. Berberine (BBH) Natural antibacterial agent Epidermal growth factor (EGF)	Wound dressings
[41]	ALG-BA@AM&MIC Sodium alginate (ALG) Benzene boric acid (BA) Micelles (MIC) Amikacin (AM) Hyaluronic acid (HA) Cholesterol (CHOL)	Amikacin (AM) Kill off bacteria Antibiotic Naproxen (Nap) Anti-inflammatory	Wound dressing with antibacterial properties and accelerated bacterial infections wound healing through anti-inflammatory macrophage response.
[42]	CMCS/Odex/OPC/PRP Carboxymethyl chitosan (CMCS) Oxidized dextran (Odex) Oligomeric procyanidins (OPC) Platelet-rich plasma (PRP)	Growth factors (GFs) Regulation of the complex healing process and of the cells taking part in this process. Platelet-derived growth factor (PDGF) (TGF-β1, FGF, PDGF)	Smart wound dressing
[33]	CS-HCA-ICPs Catechol-grafted chitosan (CS-HCA) Curcumin-Fe^3+^ coordination nanoparticles (Icps, Cur-Fe^3+^)	Curcumin (Cur) a Natural polyphenolic compound from turmeric, has been widely used based on its pleiotropic performances in antioxidant, anti-inflammatory, and wound-healing activities. Poor solubility in the aqueous phase	Prevention of bacterial infection, reduction of inflammation, and promotion of the aureus-infected wound repair.
[36]	N-Gel/ODex Ethylenediamine-modified gelatin (N-Gel). Oxidized dextran (ODex).	Zinc oxide nanoparticles (nZnO) Excellent biocompatibility, exhibiting superior antibacterial activity. The accumulation of nZnO can stimulate the liberation of Zn^2+^ and generation of ROS, thereby inducing DNA damage in bacteria. Paeoniflorin (Pf) Angiogenic activity without significant toxicity. Encapsulation of Pf into micelles (MIC) generated using an amphiphilic DSPE-TK-PEG2k-NH 2 copolymer	Diabetic wound treatment through a combination of hemostatic, antibacterial, and angiogenic activities.
[34]	CS/β-Ala/γ-PGA Chitosan (CS) poly(γ-glutamic acid) (γ-PGA) Amino acids (AA) (β-Ala)	Benzalkonium chlorides (BAC) Quaternary ammonium compounds (QACs) with their broad-spectrum antimicrobial properties.	Alkaline-adapted wound dressing

An injectable multifunctional based on gelatin hydrogel that responds to pH fluctuations is introduced by Zhang and colleagues (2023) [38]. Gel is crosslinked with benzaldehyde-modified Pluronic F127 (FCHO), a process that strengthens the pH responsiveness through the formation of Schiff base bonds and enables the incorporation of hydrophobic APIs in polymeric micelles. Curcumin is incorporated into the hydrogel using FCHO micelles to improve its solubility and stability. Magnesium-based micromotors (Mg-micromotors) also participate as H_2_ generators in the hydrogel matrix, aiming to reduce ROS (reactive oxygen species) levels at the wound site and enhance the therapeutic efficacy. The hydrogel degrades faster in an acidic environment (pH 5.0) favoring its usage for the healing of diabetic wounds by reducing wound inflammation and neutralizing ROS [38].

An antibacterial supramolecular hydrogel with pH sensitivity is prepared based on amphiphilic pentapeptides (APPs), which possess an amino acid sequence with a hydrophobic core, due to hydrophobic π-π stacking, and a hydrophilic end for opposite charges. At neutral pH, the APPs are biocompatible due to electrostatic interactions between the positively and negatively charged ions in the molecule. In contrast, at acidic pH, the protonation of the APP chains increases the positively charged ions, resulting in pH-dependent antibacterial action. Due to this mechanism, the hydrogel responds to pH changes, enabling the release of APIs at the acidic wound site. The thermosensitive polymer PNIPAm and the antibacterial Origanum essential oil are incorporated into the APP hydrogels. PNIPAm is used as a crosslinking agent to enhance both the structure and mechanical stability as well as to reduce the spontaneous release of APIs. The hydrogel managed to accelerate the healing process of infected wounds, providing a promising strategy for the treatment of chronic clinical wounds [39].

Bacterial growth-Induced tobramycin smart release self-healing hydrogel for *Pseudomonas aeruginosa*-infected burn wound healing was recently designed and developed [40].

The hydrogel being developed by Hu and colleagues (2020) [41] aims at the dual delivery of APIs for the amelioration of the wound healing process. Initially, the hydrogel is based on benzene boric acid (BA) in its ionized hydrophilic form, as this form can easily bind to substances containing o-diol groups through reversible covalent bonds, forming a more hydrophilic structure. These covalent bonds provide the hydrogel with dual pH/ROS responsiveness. Sodium alginate is crosslinked with BA to form the ALG-BA hydrogel base. Then, an amphiphilic polymer is synthesized by linking hyaluronic acid with cholesterol (HA-CHOL). Micelles containing the antibiotic amikacin are integrated into the ALG-BA polymer, while micelles containing the hydrophobic anti-inflammatory agent naproxen are incorporated into the HA-CHOL polymer. This dual-responsive hydrogel can release the loaded micelles when exposed to the acidic environment of the bacterial wound due to the breakdown of the ester boronic bonds [41].

Platelet-rich plasma (PRP) is a blood derivative rich in growth factors (GFs), which are essential for regulating the growth and differentiation of mesenchymal cells, as well as the production of the extracellular matrix. PRP has also been shown to prevent the body’s inflammatory response and promote angiogenesis and epithelialization, characteristics that make it an excellent choice for wound healing and tissue regeneration. The best delivery system for PRP is a multifunctional hydrogel with high porosity and swelling capability. This hydrogel is prepared using polymers with inherent antibacterial properties to avoid the use of antibiotics. For this purpose, oligomeric procyanidins that contain catechol groups and are known for their antibacterial and antioxidant properties, a water-soluble derivative of chitosan, and oxidized dextran were the selected biomaterials. When the hydrogel is exposed to the acidic environment of the wound, the Schiff base and hydrogen bonds that maintain its structure break, resulting in the controlled release of growth factors at the site [42].

### 3.3. Alternative Applications

The OPF-DOPA hydrogel is developed as a scaffold for implantation, aiming at bone regeneration, and its design is based on the underwater adhesion strategy of marine mussels. Oligo [poly(ethylene glycol) fumarate] (OPF) is a synthetic polymer that, by utilizing its unsaturated bonds and ester bonds, allows rapid degradation of the network via hydrolysis of these bonds in a physiological environment. OPF aids in bone regeneration through the incorporation of bioactive molecules, such as growth factors that facilitate osteoblast proliferation, differentiation, and the rearrangement of implants. However, its reduced adhesion capability, particularly on wet surfaces, limits its use, as it increases the likelihood of implants being displaced from the implantation site after injection. To address this issue, L-3,4-dihydroxyphenylalanine (DOPA) is incorporated into the hydrogel. DOPA has the ability to adhere to surfaces when reduced and form intramolecular covalent crosslinks when oxidized. These capabilities are directly influenced by the pH and ions of the environment. DOPA is also used to enhance the proliferation of pre-osteoblast cells [43].

Carboxymethyl chitosan and oxidized hyaluronic acid are used for the encapsulation of metformin for the treatment of breast cancer. Hyaluronic acid can bind to the CD44 receptor of cancer cells, increasing its selectivity for them, while its aldehyde groups in the oxidized state form covalent Schiff base bonds with the amino groups of metformin, achieving pH-responsive release. In this way, the targeted drug release is accomplished by the collapse of the hydrogel’s matrix under acidic conditions, which are characteristic of the tumor site [44].

A hydrogel based on hyaluronic acid can achieve long-term lubrication and mechanical dispersion, reducing friction in osteoarthritis. Platelet-rich plasma is incorporated into the hydrogel to promote cartilage formation, bone relining, and the wound-healing process. Moreover, artificial inorganic nanomaterials with intrinsic enzymatic mimetic properties, such as bovine serum albumin (BSA-MnO_2_) (BM) and Prussian blue nanoparticles (NPs), are included in the system to reduce the concentration of reactive oxygen species and the inflammatory response, which are undesirable effects of osteoarthritis. Additionally, the release of Mn^2+^ promotes chondrogenic differentiation of mesenchymal cells. Schiff base reactions between the aldehyde and amino groups of hyaluronic acid derivatives form the hydrogel network showing self-healing and pH-responsive properties. The active ingredients can be released when the hydrogel is exposed to the acidic inflammatory microenvironment due to the breakdown of the Schiff base bonds [45].

Exendin-4 (EX) is an antidiabetic API that is loaded into a hydrogel comprised of chitosan with the aim of modified release. The EX is incorporated into the biopolymer using the electrospray method, and then the pH-sensitive oligomer serine-b-poly(lactide)-b-poly(ethyleneglycol)-b-poly(lactide)-b-oligomer serine (OS-PLA-PEG-PLA-OS) pentablock copolymer is integrated. The electrostatic interactions between the cationic biopolymer and the negatively charged EX in the hydrogel can prevent the spontaneous release of EX, maintaining its therapeutic effect for a longer period compared to injectable EX administration, while the block copolymer ensures the transition from solution to gel state and vice versa, ensuring prolonged API release and reducing the need for repeated injections, along with the associated side effects [46].

**Figure 3:**
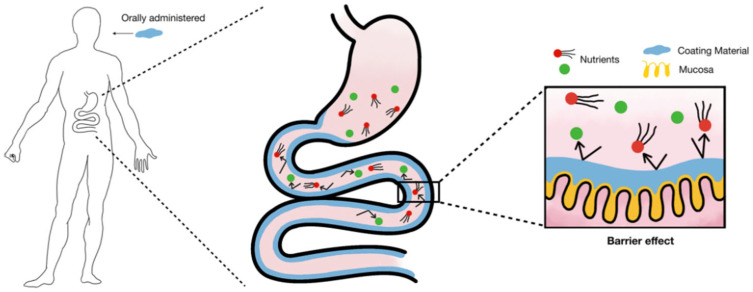
Schematic illustrations of ideas and materials used in this study. Schematic illustrations of an orally administered polyelectrolyte hydrogel as a transient barrier to inhibit excess nutrient uptake. Adapted from [47].

Pectin, a natural polysaccharide with strong adhesion capability, can form hydrogels through electrostatic interactions between multivalent cations and the deprotonated carboxyl groups of the molecule at a specific pH. A pectin-based hydrogel is utilized for the treatment of complications associated with metabolic syndromes, such as type II diabetes, obesity, and nonalcoholic fatty liver disease. The ultimate goal of this hydrogel is to limit excessive nutrient absorption without the need for invasive and irreversible procedures (e.g., Roux-en-Y gastric bypass). Therefore, sucralfate is incorporated into the hydrogel through electrostatic interactions between the deprotonated carboxyl groups of pectin and the API’s aluminum ions. The hydrogel is designed to target the mucosa of the intestine and to adhere as a barrier, limiting the absorption of excessive nutrients, as shown in Figure 3. The three-dimensional system created at pH 6.8 adheres to the intestinal mucosa and effectively prevents the reduction of the glucose response and nutrient absorption [47].

One of the most challenging therapeutic approaches is the restoration of large bone defects, which are almost always accompanied by bacterial infections and inflammation. For more effective treatment, a multifunctional hydrogel was proposed by Yao et al. (2022) [48]. The base of the hydrogel is made using gelatin methacryloyl (GelMA) and oxidized sodium alginate (OSA). GelMA contains multiple amine groups, which bind reversibly to the aldehyde groups of OSA through the formation of Schiff base bonds to create a hybrid hydrogel scaffold with enhanced mechanical stability. The Schiff base bonds break and reform in a pH-dependent manner, allowing for controlled drug release. The hydrogel co-delivers an antibiotic_gentamicin sulfate—and mesoporous silica nanoparticles loaded with phenamil, which is an activator of bone morphogenetic protein 2. The presence of bacterial infection leads to a decrease in the environmental pH (pH 4.5), which causes the breakdown of Schiff base bonds, resulting in increased swelling of the drug delivery system and enhanced API release [48].

To achieve targeted delivery and avoid side effects of chemotherapy, multifunctional hydrogels could be used. Black phosphorus nanosheets (BNPSs) have a high photothermal conversion capacity, easy fabrication, and relatively high chemical reactivity. They also have excellent biocompatibility and biodegradability. Due to these characteristics, BNPSs are used to produce hydrogels for targeted drug delivery under specific conditions. In this manner a hydrogel comprised of dibenzaldehyde-functionalized polymer, polyaspartylhydrazide polymer, and BPNSs is formulated and used for the targeted and prolonged release of doxorubicin in tumors, where, due to increased glycolysis, the pH is lower than normal. The hydrazone bonds of the hydrogel hydrolyze at low pH, releasing the API, and when the pH increases, the hydrogel regains its previous form in a reversible manner [49].

Another multifunctional hydrogel could inhibit the formation of the bacterial biofilm, a characteristic feature of chronic osteomyelitis, in combination with drug delivery of antibacterial APIs. This hydrogel shows physical stability and pH sensitivity, consisting of chitosan, gelatin, and aldehyde hyaluronic acid. In this network other nanoparticles participate as well, such as nano-TiO_2_ enhancing the crosslinks that stabilize the network, and ZIF-8, a metal-organic framework composed of metal ions connected to organic ligands that release their cargo in acidic environments while remaining stable under neutral conditions. The hydrogel also incorporates CaCl_2_ so that the Ca^2+^ that are released inhibit biofilm formation and the antibiotic vancomycin. The ZIF-8-coated hydrogel releases Ca^2+^ and vancomycin effectively due to the breakdown of Schiff base bonds under acidic conditions, which represent the environment of a chronic bacterial infection [50].

An injectable alginate-based hydrogel is designed to achieve photothermal release of Ca^2+^ in response to a specific pH, targeting the induction of cancer cell death and melanoma treatment. The hydrogel contains nanoparticles with photothermal ability due to its cargo: CaCO_3_ and polydopamine. Upon injection into the tumor site, the acidic environment breaks down the nanoparticles, enabling sustained release of Ca^2+,^ which reacts with sodium alginate chains to form hydrogels. The released Ca^2+^ ions lead to dysfunction of mitochondrial regulation and inhibition of ATP synthesis, resulting in apoptosis of cancer cells [51].

An innovative pH-responsive hydrogel is developed by Pourmadadi et al. (2024) [52] using a polymer comprised of polyethylene glycol and agarose as its base. This base ameliorates the API’s stability and enables controlled and targeted release of the incorporated API. Ferric oxide (Fe_2_O_3_) is used to improve the incorporation of 5-fluorouracil (5-Fu), commonly used for breast cancer treatment. The hydrogel exhibits a significant amount of 5-Fu release under acidic conditions (pH 5.4). This hydrogel seems like a promising drug delivery system for anticancer therapy, enhancing the API’s efficacy while reducing unwanted systemic side effects [52].

## 4. Conclusions

As mentioned in the Section 2, recent advances in pH-responsive hydrogels are the main focus of this literature review manuscript. However, it is important to note that several research groups have been working in this field for the past 30 years [53,54,55,56,57,58]. The group of Dar et al. has investigated the design and functionalization of pH-responsive polysaccharide-based hydrogels for tissue-specific drug release. Their study explores the preparation of biocompatible hydrogels with tunable swelling and degradation properties with drug release to the acidic microenvironments [53,54]. In the same context, the Hennink group has prepared and developed block copolymer-based hydrogels that exhibit reversible sol–gel transitions and high responsiveness to pH alterations. Their studies provide insights into the fine-tuning of hydrogel architecture for improving the physicochemical stability of loaded APIs and their therapeutic effectiveness and safety [55,56]. Polyanhydride-Releasing Oral MicroParticle Technology (PROMPT) was designed and developed by Peppas’s group using the pH-triggered antisolvent precipitation technique. Additionally, core–shell microcapsules based on the polyanhydride poly(bis(2-carboxyphenyl)adipate) as a shell were formulated for pH-controlled triggered release using the hydrophobic probe pyrene as a model API [57,58].

Furthermore, the type and the chemical characteristics of the used polymer are found to play a key role in the final conformation of the pH-responsive hydrogels, as well as in their later behavior as drug delivery systems. The ability of flexible customization of the polymeric derivatives leads to a great variety of different hydrogel candidates of various functionalities being combined with other polymers, either natural or synthetic, or other functional biomaterials, such as embedded nanoparticles. Taking into account the above-described literature examples, the type of the ionizable groups of the pH-responsive polymers, the pH values exhibiting the response, their crosslinking mode, their water solubility, swelling capacity, porosity, and mechanical strength can be controlled during the formulation process upon the formation of final hydrogel systems with desirable characteristics as regards their drug release kinetics, targeting ability, hydrogel’s stability, drug-loading efficiency, and improved therapeutic effectiveness with parallel minimized side effects. Different pharmaceutical applications have been presented in the above paragraphs, including drug delivery, wound healing, mucoadhesive systems, anticancer therapy, oral administration, matrixes, tissue regeneration, applications against bacterial infection from biofilm formation, and many other examples, highlighting the great potential of the pH-responsive hydrogels in the field of therapeutics. In conclusion, pH hydrogels are platforms for several applications in biomedical sciences. The drug delivery and targeting applications, as well as wound-healing properties, useful in the design and development of medical devices, make pH-responsive hydrogels potential candidates for fast clinical translation. The improved biodegradability and the multifunctional targeting strategies are the most important critical quality attributes for several future perspectives in pharmaceutics. On the other hand, the variability of several biopolymers, the main ingredients of hydrogels, and the scale-up limitations accompanied by several techniques for their evaluation in biological systems are the main challenges for pharmaceutical technology. Having all the above in mind, multidisciplinary scientific input is needed to increase the impact of pH-responsive hydrogels in pharmaceutical applications.

## Abbreviations

The following abbreviations are used in this manuscript:

AA	Acrylic acid
A-HA	Aldehyde hyaluronic acid
AMPS	2-acrylamido-2-methylpropane-sulfonic acid
API	Active Pharmaceutical Ingredient
APPs	Amphiphilic pentapeptides
BA	Boric acid
BC	Bacterial cellulose
BM	Bovine serum albumin (BSA-MnO2)
BNPSs	Black phosphorus nanosheets
CA	Citric acid
CE	CA-EPL
CEC	N-carboxyethyl chitosan
CMCS	Carboxymethyl chitosan
CS	Chitosan
CS/GP	Chitosan and Glycerophosphate
DA	Dihydrocaffeic acid
DF-PEG-PAHy/BPNSs	Dibenzaldehyde-functionalized polymer (DF-PEG) and polyaspartylhydrazide (PAHy) polymer
DOP	Dopamine
DOPA	L-3,4-dihydroxyphenylalanine
DOX	Doxorubicin
EPL	ε-polylysine
EX	Exendrin-4
FCHO	Pluronic F127
Fe_2_O_3_	Ferric oxide
Gel	Gelatin
GelMA	Gelatin methacryloyl
GFs	Growth factors
GO	Graphene oxide
HA	Hyaluronic acid
HA-CHOO	Hyaluronic acid-aldehyde
HA-CHOL	Hyaluronic acid with cholesterol
HZ-Alg	Hydrazine-functionalized alginate
IA	Itaconic acid
LCST	Lower critical solution temperature
MF	Mangiferin
MIC	Micelles
MSCC	Millettia speciosa Champ cellulose
MSCCMC	M. speciosa carboxymethyl cellulose
NIR	Near-Infrared
NPs	Prussian blue nanoparticles
NSA	Nitrosalicylaldehyde
OAL	O-allyl chitosan
OD	Oxidized dextran
OGA	Polyaldehyde gum Arabic
OHA	Oxidized Hyaluronic Acid
OP	Oxidized Pullulan
OPF	Oligo [poly(ethylene glycol) fumarate]
OSA	Oxidized sodium alginate
OS-PLA-PEG-PLA-OS	oligomer serine-b-poly(lactide)-b-poly(ethyleneglycol)-b-poly(lactide)-b-oligomer serine
PAG	Prunus armeniaca gum
PBA	Phenylboronic acid
PEG	Polyethylene glycol
PEGS-BA	Benzaldehyde-functionalized polyethylene glycol-co-poly(glycerol sebacic acid)
PEG-SH	Four-arm polyethylene glycol
PEI	Polyethyleneimine
pH-GSH	pH/glutathione
PMB	Polymyxin B
PRP	Platelet-rich plasma
PUE	Puerarin
PVA	Poly(vinyl alcohol)
rGO@PDA	Polydopamine-coated reduced graphene oxide
ROS	Reactive oxygen species
SGDA	Succinoglycan dialdehyde
SGDA/ACD	Succinoglycan dialdehyde/aminoethylcarbamoyl-β-cyclodextrin
TCS	Thiolated chitosan
